# Supplementary material for: An Isolate of Streptococcus mitis Displayed In Vitro Antimicrobial Activity and Deleterious Effect in a Preclinical Model of Lung Infection
Source: Nutrients. 2023 Jan 4;15(2):263. doi: 10.3390/nu15020263 (PMC9867278; doi:10.3390/nu15020263)
Supplement: Supplementary file 1 [file nutrients-15-00263-s001.zip › nutrients-2043456-supplementary.pdf]

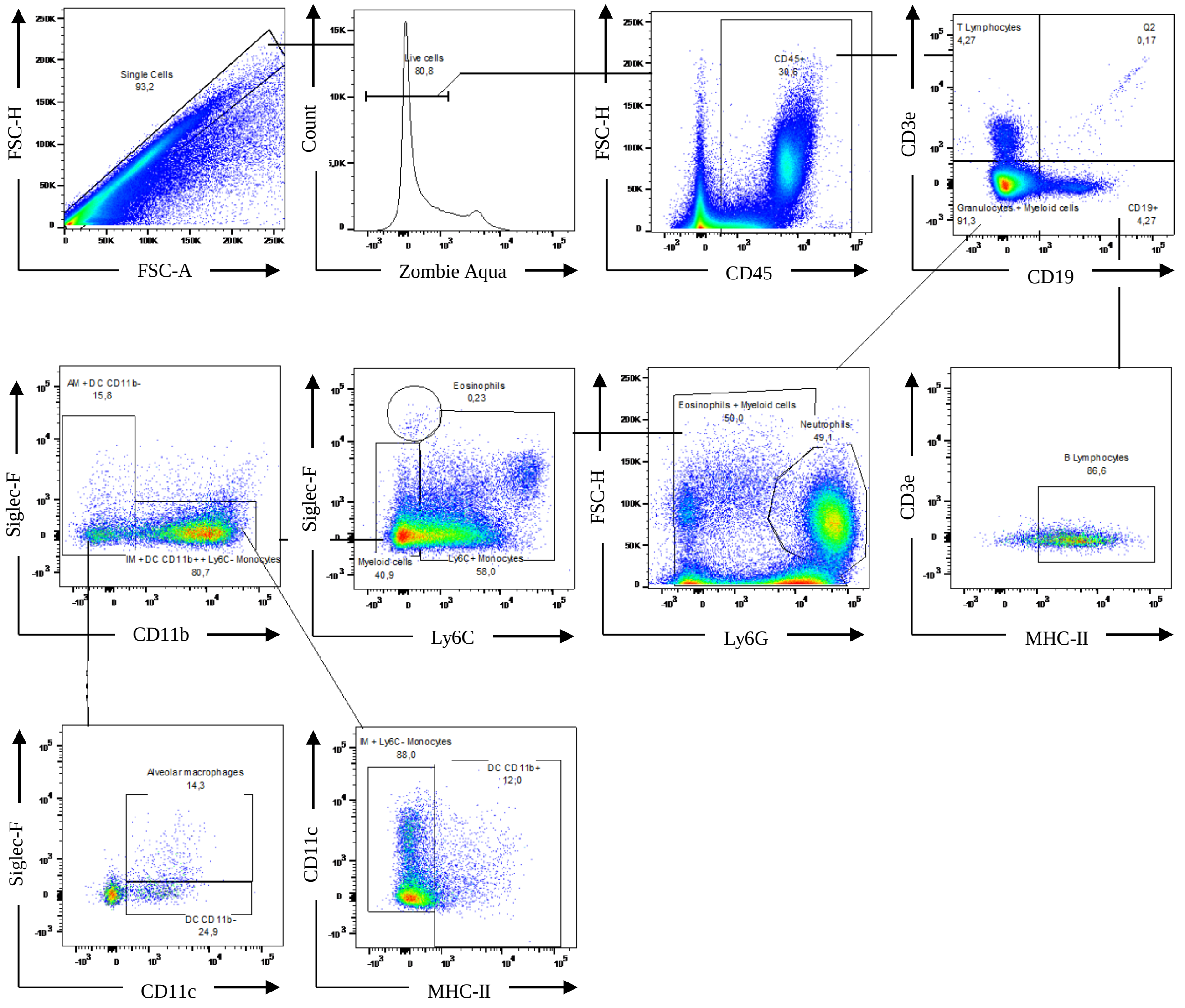

Figure S1: Gating strategy. The frequency of immune cells were determined in lung tissue 48 hours post-infection.

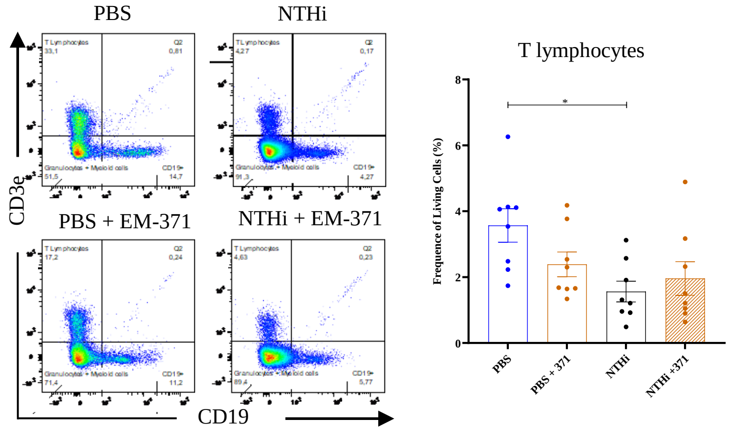

Figure S2: T lymphocytes were negatively regulated in infected mice. The frequency of lymphocytes T were determined in lung tissue 48 hours post-infection. Value are expressed as the mean ± s.e.m. for one experiment (8 mice/group). To determine the statistical difference, One-way ANOVA using Tukey multiple comparisons test was performed with GraphPad version 8. *p*-values: *: $p<0.03$.
